# Supplementary material for: Time-delay model of perceptual decision making in cortical networks
Source: PLoS One. 2019 Feb 15;14(2):e0211885. doi: 10.1371/journal.pone.0211885 (PMC6377186; doi:10.1371/journal.pone.0211885)
Supplement: S1 File — (PDF) [file pone.0211885.s001.pdf]

S1 Stability analysis of the model

Consider the system of equations

$$\begin{aligned} \dot{r}_1 &= \alpha\Big(I - r_1(t-\tau) + \epsilon r_2 f(r_1 r_2)\Big), \\ \dot{r}_2 &= \alpha\Big(I - r_2(t-\tau) + \epsilon r_1 f(r_1 r_2)\Big), \end{aligned} \tag{1}$$

where $\alpha = \frac{1}{\tau_w}$ is a time scale, I is a constant input and $f(x) = \frac{x^2}{1+x^2}$. We are mainly interested in the model dynamics for reference parameter values

$$I = 0.4, \quad \epsilon = 1.$$

On the other hand, as the qualitative dynamics of Eqs. (1) does not depend on the magnitude of α, in the following we assume $\alpha = 1$.

Looking for steady states we obtain

$$-\bar{r}_1 + \epsilon\bar{r}_2 f(\bar{r}_1\bar{r}_2) = -\bar{r}_2 + \epsilon\bar{r}_1 f(\bar{r}_1\bar{r}_2),$$

where $(\bar{r}_1, \bar{r}_2)$ denotes the steady state. This relation implies

$$\bar{r}_2 - \bar{r}_1 = \epsilon(\bar{r}_1 - \bar{r}_2) f(\bar{r}_1\bar{r}_2) \quad \Longrightarrow \quad \bar{r}_1 = \bar{r}_2 = \bar{r},$$

as $f(x) \neq -1/\epsilon$ due to non-negativity of this function. Therefore, the coordinate $\bar{r}$ satisfies

$$I = \bar{r} - \epsilon\frac{\bar{r}^5}{1+\bar{r}^4}.$$

Let us consider an auxiliary function defined as $g_\epsilon(r) = r\frac{1+r^4(1-\epsilon)}{1+r^4}$, and for the reference $\epsilon = 1$ this function reduces to $g_1(r) = \frac{r}{1+r^4}$. It is easy to see that g_1 is unimodal, $g_1(0) = 0$, $\lim_{r\to\infty} g_1(r) = 0$, and has its maximal value $\frac{3^{3/4}}{4}$ at $r = 3^{-1/4}$. This means that there are at most two steady states, depending on the value of $I > 0$. On the other hand, if $\epsilon \neq 1$, then the derivative $g'_\epsilon(r)$ could have two positive zeros, and therefore there are at most three steady states. For the reference $I = 0.4$, let us consider another auxiliary function $h(r) = 1 - \frac{0.4r^4 - r + 0.4}{r^5}$. There is $\lim_{r\to\infty} h(r) = 1$ and $\lim_{r\to 0} h(r) = -\infty$. Moreover, $h(r) > 0$ for $r > 0.4$ and we can check that $h'(r)$ has two zeros in the interval $(0, 2)$, around 0.5065858562 (for which there is maximum of h around 3.405131243) and 1.952080163 (for which there is minimum of h around 0.84984565). Hence, there are at most three steady states, depending on the magnitude of ϵ.

Corollary 0.1. *If $\epsilon = 1$, then*

- *for $I < \frac{3^{3/4}}{4}$ there are two steady states of Eqs.* (1)*;*
- *for $I = \frac{3^{3/4}}{4}$ there is exactly one steady state of Eqs.* (1)*;*
- *for $I > \frac{3^{3/4}}{4}$ there is no steady state of Eqs.* (1).

If $I = 0.4$, then

- *for $\epsilon < 0.84984565$ and $\epsilon \approx 3.405131243$ there is exactly one steady state of Eqs.* (1)*;*
- *for $\epsilon \approx 0.84984565$ and $1 \leq \epsilon < 3.40513124$ there are two steady states of Eqs.* (1)*;*

- *for* $0.84984565 < \epsilon < 1$ *there are three steady states of Eqs.* (1)*;*
- *for* $\epsilon > 3.405131243$ *there is no steady state of Eqs.* (1).

Remark 0.2. *For the reference parameter values* $\epsilon = 1$ *and* $I = 0.4$ *there are two steady states of Eqs.* (1)*, around* 0.4114655 *and* 1.1827404.

Looking for local stability of these states for $\tau = 0$ we calculate Jacobi matrix for Eqs. (1) obtaining

$$MJ(r_1, r_2) = \begin{pmatrix} -1 + \epsilon r_2^2 f'(r_1 r_2) & \epsilon f(r_1 r_2) + \epsilon r_1 r_2 f'(r_1 r_2) \\ \epsilon f(r_1 r_2) + \epsilon r_1 r_2 f'(r_1 r_2) & -1 + \epsilon r_1^2 f'(r_1 r_2) \end{pmatrix},$$

and for a steady state $(\bar{r}, \bar{r})$ it reads

$$MJ(\bar{r}, \bar{r}) = \begin{pmatrix} -1 + \eta & \beta + \eta \\ \beta + \eta & -1 + \eta \end{pmatrix},$$

where $\eta = \epsilon \bar{r}^2 f'\left(\bar{r}^2\right) >$ and $\beta = \epsilon f\left(r^2\right) > 0$.

We have $\operatorname{tr} MJ(\bar{r}, \bar{r}) = -2 + 2\eta$ and $\det MJ(\bar{r}, \bar{r}) = 1 - 2\eta - 2\eta\beta - \beta^2$. Hence, the necessary condition of stability is $\eta < 1$, that is $f'\left(\bar{r}^2\right) < \frac{1}{\epsilon \bar{r}^2}$. Moreover, calculating the determinant of the characteristic equation we easily see that it is always positive, such that any steady state is either a saddle or a node.

For $\bar{r} \approx 0.4114655$ we have $\det MJ(\bar{r}, \bar{r}) \approx 0.7862262548$ and $\operatorname{tr} MJ(\bar{r}, \bar{r}) \approx -1.891645542$, which means that this point is a stable node. For $\bar{r} \approx 1.1827404$ we have $\det MJ(\bar{r}, \bar{r}) \approx -0.5901784196$, which means that this state is a saddle.

Corollary 0.3. *For the reference parameter values* $I = 0.4$ *and* $\epsilon = 1$ *the steady state with smaller coordinates is a stable node, while the steady state with greater coordinates is a saddle.*

Moreover, analyzing the phase space portrait we are able to check that all solutions below the stable manifold of the saddle are attracted by the stable node, while above this manifold all solutions go to infinity.

Now we turn to the case $\tau > 0$. We know that if a steady state is a saddle for $\tau = 0$ then it remains unstable for all $\tau > 0$. Hence, we want to check if stability switches are possible for the state $(\bar{r}, \bar{r})$, $\bar{r} \approx 0.4114655$. Calculating the characteristic matrix one gets

$$\Delta(\lambda, \tau) = \begin{pmatrix} -\exp(-\lambda\tau) + \eta - \lambda & \beta + \eta \\ \beta + \eta & -\exp(-\lambda\tau) + \eta - \lambda \end{pmatrix}.$$

To get stability switches one needs to find eigenvalues $\lambda = \pm i\omega$, $\omega > 0$, in the imaginary axis and check if they cross this axis.

Let us take $\lambda = i\omega$. Then

$$\det \Delta(i\omega, \tau) = (-\exp(-i\omega\tau) + \eta - i\omega)^2 - (\beta + \eta)^2 = \left(i\omega + \mathrm{e}^{-i\omega\tau} + \beta\right)\left(i\omega + \mathrm{e}^{-i\omega\tau} - \beta - 2\eta\right).$$

It is obvious that $\det \Delta(i\omega, \tau) = 0$ iff $W_1(i\omega, \tau) = i\omega + \mathrm{e}^{-i\omega\tau} + \beta = 0$ or $W_2(i\omega, \tau) = i\omega + \mathrm{e}^{-i\omega\tau} - \beta - 2\eta = 0$. As W_1 and W_2 are very well know transcendental equations, we can conclude that

1. if $\beta + 2\eta < 1$, then there are two sequences of critical delays $\left(\tau_n^1\right)$ for W_1 and $\left(\tau_n^2\right)$ for W_2 at which stability switches are possible;
2. if $\beta < 1$ and $\beta + 2\eta > 1$, then there is only one sequence of critical delays corresponding to W_1;
3. if $\beta > 1$, then stability switches are not possible.

It occurs that for the reference parameter values $I = 0.4$ and $\epsilon = 1$ the first case occurs. Moreover, we know that for the first critical delay the stable steady state loses stability and cannot gain it again. This means that we can suspect that solutions of Eqs. (1) oscillates permanently for sufficiently large delays.
