# Supplementary material for: Time-delay model of perceptual decision making in cortical networks
Source: PLoS One. 2019 Feb 15;14(2):e0211885. doi: 10.1371/journal.pone.0211885 (PMC6377186; doi:10.1371/journal.pone.0211885)
Supplement: S2 File — (PDF) [file pone.0211885.s002.pdf]

S2 The Balloon-Windkessel model of the hemodynamic response function

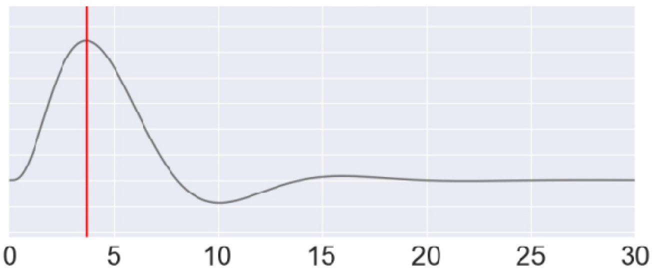

S2 Fig A The canonical HRF response ($\kappa = 0.65[1/s]$, $\gamma = 0.41[1/s]$, $\lambda = 0.98[s]$, $\alpha = 0.32$, $\rho = 0.34$).

The classic Balloon-Windkessel model for the hemodynamic response [1, 2] reads as follows

$$\begin{cases} \dot{s}(t) & = & r(t) - \kappa s(t) - \gamma(f(t) - 1), \\ \dot{f}(t) & = & s(t), \\ \lambda\dot{v}(t) & = & f(t)v(t)^{1/\alpha}, \\ \lambda\dot{q}(t) & = & f(t)E(f(t), \rho)/\rho - v^{1/\alpha-1}(t)q(t), \end{cases} \tag{1}$$

where $r(t)$ – the underlying, fast neuronal dynamics, $s(t)$ – vasodilatory signal, $f(t)$ – inflow, $v(t)$ – blood volume, $q(t)$ – deoxyhemoglobin content, $E(f, \rho) = 1 - (1 - \rho)^{1/f}$. This model contains five node-specific constants: κ – rate of signal decay, γ – rate of flow-dependent elimination, λ – hemodynamic transit time, α – Grubb's exponent, ρ – resting oxygen extraction fraction.

Then, the following expression describes the BOLD response

$$y(t) = V_0(7\rho_i(1 - q(t)) + 2(1 - q(t)/v_i(t)) + (2\rho - 0.2)(1 - v(t))), \tag{2}$$

where $V_0 = 0.02$ is the resting blood volume fraction. The hemodynamic parameters were set at the mean of the distributions given in [2]: $\kappa = 0.65[1/s]$, $\gamma = 0.41[1/s]$, $\lambda = 0.98[s]$, $\alpha = 0.32$, $\rho = 0.34$.

This generative model characterizes BOLD response $y(t)$ in a function of fast neuronal dynamics $r(t)$ as a system of ordinary differential equations. Effectively, $y(t)$ can also be obtained from $r(t)$ as a linear convolution with a kernel which describes BOLD response to a pulse stimulus $r(t)$, also known as a hemodynamic response function (Fig A).

References

1. Buxton RB, Wong EC, Frank LR. Dynamics of blood flow and oxygenation changes during brain activation: the Balloon model. Magnetic Resonance in Medicine. 1998;39(6):855–64. doi:10.1002/mrm.1910390602.

2. Friston KJ, Harrison L, Penny W. Dynamic Causal Modeling. NeuroImage. 2003;19(4):1273–302. doi:10.1016/S1053-8119(03)00202-7.
