# Supplementary material for: Time-delay model of perceptual decision making in cortical networks
Source: PLoS One. 2019 Feb 15;14(2):e0211885. doi: 10.1371/journal.pone.0211885 (PMC6377186; doi:10.1371/journal.pone.0211885)
Supplement: S3 File — (PDF) [file pone.0211885.s003.pdf]

S3 Alternative scenarios

Fixing synaptic weights

Let us remind the model equations:

$$\dot{r}_1 = \alpha\Big(I - r_1(t-\tau) + \epsilon r_2 f(r_1 r_2)\Big),$$
$$\dot{r}_2 = \alpha\Big(I - r_2(t-\tau) + \epsilon r_1 f(r_1 r_2)\Big), \tag{1}$$

In our model, synaptic weights are plastic (i.e. we have Hebbian plasticity). Alternatively, we could consider a model with fixed synaptic weights. Fixing synaptic weights effectively means fixing the Hill function $f(r_1(t)r_2(t))$ in Eq. (1). Therefore, we performed additional simulations, exchanging the Hill function $f(r_1(t)r_2(t))$ with two constants:

1. a constant $f(r_1^*, r_2^*) = 0.027$, which is in the same range of magnitude as the range in which Hill function $f(r_1(t)r_2(t))$ was fluctuating before. In a result, stationary points in the model are close to what we had before, namely $(r*1, r*2) = (0.4111, 0.4111)$ as opposed to the previous value $(r_1^*, r_2^*) = (0.411466, 0.411466)$.
2. a constant $f(r_1(t)r_2(t)) = 0.2$, which is an order of magnitude higher than the range in which Hill function $f(r_1(t)r_2(t))$ was fluctuating before. In a result, the new stationary points in the model are $(r_1^*, r_2^*) = (0.5, 0.5)$.

In general, the dynamics is similar as in case of plastic synapses, as the major difference comes from shifting the stability points. In some sub-bifurcation cases (marked in Fig. A in red), fixed weights lead to longer oscillations in probabilities than plastic synapses, but in general it is only quantitative and not qualitative difference.

Additional terms in the model

The model introduced in Eq. (1) could be extended in two ways, introduced below.

1. when next to the delayed inhibition, we add *instantaneous relaxation* (i.e., self-inhibition without delay). The influence of adding the instantaneous relaxation term could be explained on the following, simplified example.

   What generates oscillations in this network, is the relaxation term. Therefore we can consider the dynamics including two relaxation terms instead of one (delayed and instantaneous), and skipping the term characterizing the influence from the other population:

   $$\dot{r}(t) = a(I - r(t-\tau) - br(t)), \tag{2}$$

   where $b > 0$.
   (in the manuscript, $b = 0$). In this case, the dynamics depends on the value of parameter b. One can find detailed analysis of this equation in the classic textbook dedicated to delayed differential equations by [1].

   In short, there are three possible cases:

   - if $b > 1$, then the dynamics is governed by the term without delay: there will be no stable oscillations as the system will always be attracted to the stationary point.

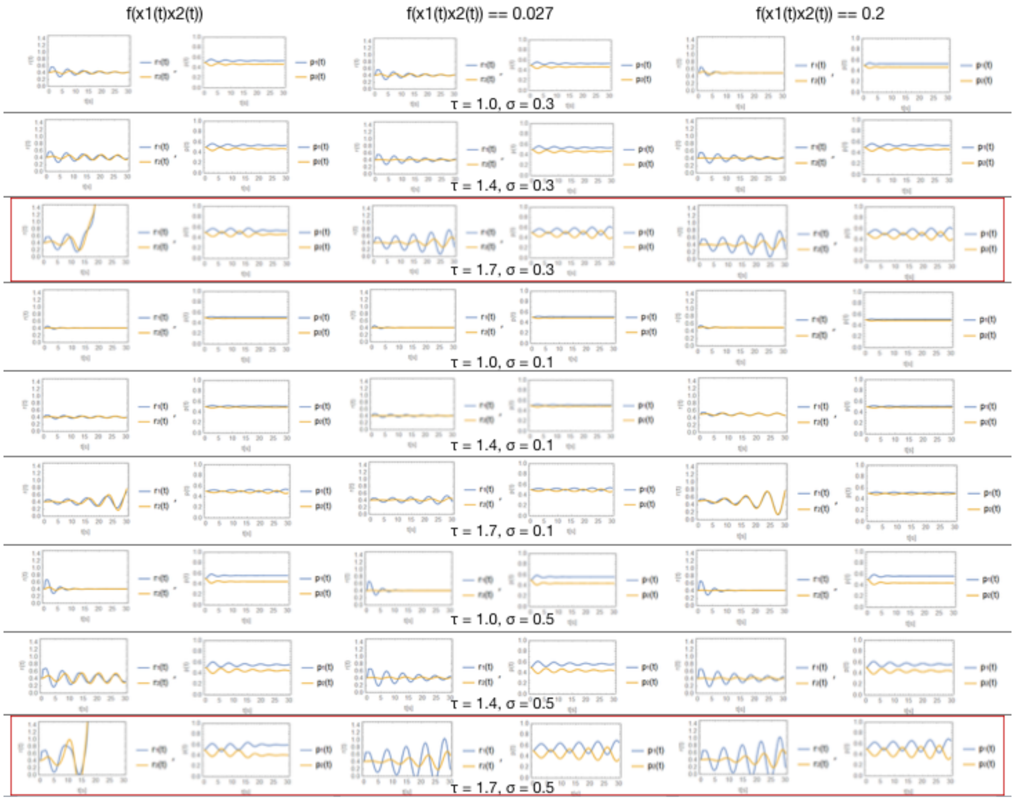

S3 Fig A Plastic synaptic weights versus fixed synaptic weights. In general, the dynamics is similar as in case of plastic synapses, as the major difference comes from shifting the stability points. In some sub-bifurcation cases (marked in red), fixed weights lead to longer oscillations in probabilities than plastic synapses, but in general it is only quantitative and not qualitative difference.

- if $0 < b < 1$, then the dynamics is governed by the term with delay and we have oscillations similarly as in case $b = 0$. Therefore, the system behaves the same as the system analyzed in the manuscript in qualitative terms, and the difference is only quantitative as the stationary points change.
- in the particular case of $b = 1$, the dynamics is more complex, see [1].

2. when we add *self-excitation* to the model, which corresponds to the following equation:

$$\dot{r}(t) = a(I - r(t-\tau) + br(t)), \tag{3}$$

where $b > 0$. In this case, the dynamics again depends on the value of parameter b:

- if $b > 1$, then the system is unstable regardless of the delayed term
- if $0 < b < 1$, then the dynamics is governed by the term with delay and we have oscillations similarly as in case $b = 0$. Therefore, the system behaves the same as the system analyzed in the manuscript in qualitative terms, and the difference is only quantitative as the stationary points change.
- in the particular case of $b = 1$, the dynamics is more complex, see [1].

References

1. Hale JK. Theory of Functional Differential Equations. Springer; 1977.
